# Quantitative effects of sex hormones on hair growth and patient satisfaction

**DOI:** 10.1210/clinem/dgag139

**Published:** 2026-04-08

**Authors:** Romy W P M de Kroon, Juliette Strating, Annemieke C Heijboer, Martin den Heijer

**Affiliations:** Department of Laboratory Medicine, Amsterdam University Medical Center, Location University of Amsterdam, Endocrine Laboratory, Meibergdreef 9, 1105 AZ, Amsterdam, The Netherlands; Amsterdam Gastroenterology, Endocrinology & Metabolism, Amsterdam, The Netherlands; Department of Laboratory Medicine, Amsterdam University Medical Center, Location University of Amsterdam, Endocrine Laboratory, Meibergdreef 9, 1105 AZ, Amsterdam, The Netherlands; Department of Laboratory Medicine, Amsterdam University Medical Center, Location University of Amsterdam, Endocrine Laboratory, Meibergdreef 9, 1105 AZ, Amsterdam, The Netherlands; Amsterdam Gastroenterology, Endocrinology & Metabolism, Amsterdam, The Netherlands; Department of Laboratory Medicine, Amsterdam University Medical Center, Location Vrije Universiteit, Endocrine Laboratory, De Boelelaan 1117, 1081 HV, Amsterdam, The Netherlands; Amsterdam Gastroenterology, Endocrinology & Metabolism, Amsterdam, The Netherlands; Department of Internal Medicine, Division of Endocrinology, Amsterdam University Medical Center, Location Vrije Universiteit, De Boelelaan 1117, 1081 HV, Amsterdam, The Netherlands; Center of Expertise on Gender Dysphoria, Amsterdam University Medical Center, De Boelelaan 1117, 1081 HV, Amsterdam, The Netherlands

**Keywords:** transgender care, hirsutism, digital microscopy, terminal hair growth

## Abstract

**Background:**

Gender-affirming hormone therapy (GAHT) alters hair distribution, a key aspect of gender transition. Transgender women often seek hair reduction, while transgender men typically desire increased hair growth. Previous studies relied on subjective hair assessments, with limited understanding of psychosocial effects.

**Objectives:**

To investigate changes in terminal hair growth in transgender men and women (both lasered and non-lasered) using digital microscopy, and their relation to body image during the first 12 months of GAHT.

**Methods:**

In this longitudinal study, 36 participants (20 transgender men, 16 transgender women) initiating GAHT were assessed at baseline, and after 3 and 12 months of GAHT. Hair densities were measured using digital microscopy. Psychosocial outcomes were evaluated using the Body Appreciation Scale-2 and satisfaction questionnaires.

**Results:**

After 12 months, transgender women showed significant reductions in hair density on the upper lip (−17 hairs/cm^2^, CI −32, −2), chin (−28 hairs/cm^2^, CI −47, −8), and upper abdomen (−6 hairs/cm^2^, CI −12,0), with no significant differences between lasered and non-lasered women. Transgender men exhibited significant increases on the upper lip (+21 hairs/cm^2^, CI 11-30), chin (+13 hairs/cm^2^, CI 3-24), upper (+17 hairs/cm^2^, CI 8-23) and lower abdomen (+20 hairs/cm^2^, CI 11-28), and thighs (+14 hairs/cm^2^, CI 7-21). Both groups reported improved body appreciation, satisfaction with hair growth, and gender congruence.

**Conclusion:**

GAHT induces measurable, location-specific changes in terminal hair growth in transgender women and men, accompanied by improvements in body image and satisfaction. Digital microscopy provides a valuable tool for future studies comparing GAHT regimens.

Body hair may be associated with significant personal distress and carries substantial cultural and societal implications (1). The perception of body hair remains largely aligned with dominant Western beauty ideals, which continue to valorize hairlessness as a marker of femininity and desirability in women (2). Traditional masculinity, on the other hand, has often embraced body hair as a symbol of virility and masculinity (3). This normative aesthetic is in direct contradiction to the core values of societal shifts toward body positivity, feminism, and the acceptance of transgender and gender-diverse identities.

Gender dysphoria refers to the feeling of discomfort or distress in people whose gender identity differs from their sex assigned at birth, or sex-related characteristics. Transgender individuals, comprising approximately 0.1-2.0% of the general population (4), may seek gender-affirming hormone therapy (GAHT), either masculinizing hormone therapy (MHT) or feminizing hormone therapy (FHT) to align their physical characteristics with their gender identity. GAHT leads to changes in body composition and secondary sex characteristics, including body hair changes. Hair distribution is a significant aspect in gender transition (5, 6), as excessive body hair has been demonstrated to contribute to gender dysphoria in transgender women by reinforcing male secondary sex characteristics (7). For these individuals, the presence of male-pattern hair, defined as hirsutism, can hinder their ability to express their female identity and negatively impact social interactions. Conversely, increased body hair is often desired in transgender men and can be a source of gender euphoria instead.

Androgens promote terminal hair growth by increasing the hair diameter, growth rate, length, and pigmentation by binding to the androgen receptor (AR) within the dermal papilla (8). This stimulates the transformation of fine, unpigmented vellus hair into thicker, pigmented terminal hair in androgen-sensitive skin areas. As a result, MHT induces androgenic hair growth in transgender men, while FHT, particularly through anti-androgens, reduces androgenic hair growth by blocking the AR, reducing the androgen production, and/or inhibiting the 5α-reductase enzyme in transgender women (9). Another component of FHT consists of estradiol supplementation, yet the effect of estradiol on hair growth remains unclear. In addition to hormonal interventions, transgender women may use laser hair removal or electrolysis is to additionally reduce hirsutism (9).

Changes in terminal hair growth are among the most visible and significant effects of GAHT, especially for individuals seeking physical alignment with their gender identity. However, hair growth is often assessed in a rather subjective way. The modified Ferriman-Gallwey (mFG) score is a widely used tool to standardize the evaluation of androgen-sensitive hair growth (10). The mFG score assesses 9 body areas, including the upper lip, chin, chest, upper and lower abdomen, upper arms, upper and lower back, and thighs. Hair growth is rated from 0 (ie, no androgenic hair growth) to 4 (ie, extensive androgenic hair growth) in each of the 9 locations. Consequently, the mFG score ranges from a minimum score of 0 to a maximum score of 36. Although the mFG provides a standardized and widely accepted method of assessing terminal hair growth, it is still subjective and lacks precision in capturing nuanced changes, as it is based on visual inspection (11-13). To complement this, recent research has introduced a more objective method using digital microscopy to quantify terminal hair (14). This method has demonstrated minimal inter-observer variability and allows for more precise measurement of hair thickness and hair density.

Alternations in terminal hair growth have previously been described solely using the mFG score, and the psychological impact of body hair changes, such as satisfaction with hair growth and overall body image, remains less understood. Therefore, this study aims to quantify changes in hair density after 3 and 12 months of GAHT in transgender men and women (both lasered and non-lasered), and to explore how transgender individuals undergoing treatment subjectively experience these physical changes.

## Methods

This longitudinal study was performed at the Center of Expertise on Gender Dysphoria, Amsterdam University Medical Center, location VUmc. Ethical review was performed by the non-WMO Review Committee. The committee determined that the study is not subject to the Dutch Medical Research Involving Subjects Act (WMO) and granted a waiver for statutory WMO approval, following a positive ethical assessment.

### Study population

All participants were included at the Center of Expertise on Gender Dysphoria at the Amsterdam University Medical Center between November 2023 and January 2025. Patients were approached when they were eligible for hormone treatment and were consecutively enrolled if they met the inclusion criteria. Inclusion criteria were a diagnosis of gender dysphoria (DSM IV or V), identification as either transgender man or woman, and initiation of GAHT as part of standard clinical care. Exclusion criteria consisted of age < 17 years and > 40 years, individuals that previously received GAHT, transgender women who performed epilation (ie, plucking or waxing), individuals that were going to start with low-dose GAHT, individuals that were hypersensitive to sunlight, had artificial intraocular lenses following cataract surgery and individuals that were motorically restless and/or had difficulty following instructions. Moreover, individuals were excluded when follow-up measurements at *t* = 1 and *t* = 2 were not available. During FHT, permanent hair removal methods, such as laser or electrolysis treatment, were not excluded in this study as these methods are frequently applied in transgender women to support the reduction of hair growth by FHT. Informed consent was obtained from all participants prior to inclusion.

### Materials

The Dino-Lite Edge AM4115T-FVW digital handheld microscope camera was used to obtain photos of the androgen-sensitive areas. This camera has a resolution of 1.3 M pixels and enables magnification between 20 and 220 times. Although the camera is equipped with both visible light and UV fluorescence LEDs (400-450 nm), we only used visible light LEDs in this study, since earlier studies showed that visible light offered the optimal way to distinguish between terminal and vellus hair (14). The assisting DinoCapture software was used on a Windows laptop to capture and analyze the photos.

### Procedures

The measurements were performed at baseline (*t* = 1) and after 3 (*t* = 1) and 12 (*t* = 2) months of using GAHT. Participants underwent physical examination, including measurement of height, weight, and body mass index (BMI) as part of standard clinical care at *t* = 0, *t* = 1, and t = 2. Afterwards, participants underwent an examination of the androgenic hair growth pattern using a digital microscope camera on the upper lip, chin, chest, upper and lower abdomen, upper arm, upper and lower back, and thighs. Due to frequent use of permanent hair removal methods during the transition, transgender women were asked to describe the number of previous laser treatment sessions at *t* = 1 and *t* = 2. Additionally, testosterone and estradiol concentrations were measured by liquid chromatography tandem–mass spectrometry as part of standard clinical care at *t* = 0, *t* = 1, and *t* = 2 (15-17).

The photos of the transgender men and women were assessed by one researcher performing a single quantification. The analysis encompassed 2 specific hair parameters: hair thickness and the number of terminal hairs per photo (ie, hair density). For each individual photo, the DinoCapture software was used to perform line measurements across individual hair shafts to assess the hair thickness of individual hairs. A cutoff value of 60 µm was used to quantify terminal hairs (14). If the diameter exceeded 60 µm, the hair was included in the terminal hair count (ie, hair density). Hairs with a diameter < 60 µm, ingrown hairs, or hairs that barely reached the skin's surface were excluded from the analysis. Non-responders were defined as having <10 hairs/cm^2^ on the face (eg, upper lip and chin).

After the physical examination, participants completed a questionnaire. The 10-item Body Appreciation Scale-2 (BAS-2) was the first part of the questionnaire and was used to assess individuals' acceptance of, favorable opinions toward, and respect for their bodies (18). Moreover, the impact of terminal hair growth was investigated by a questionnaire examining the satisfaction of individuals' current hair growth pattern by a 5-point Likert scale ranging from “Very Satisfied” to “Very Dissatisfied” per location of the mFG score. Moreover, the following items were used to further explore cognitions, emotions, and behaviors toward hair growth: (1) “I feel insecure about the hair growth on by body”, (2) “I experience the extent of body hair as distressing,” (3) “I worry about the extent of hair on my body,” and (4) “I perceive the amount of my body hair as congruent with my gender identity.”

### Power analysis

The sample size was determined based on the expected agreement for the assessment of hirsutism, defined as an mFG-score ≥ 8, as observed in the European Network for the Investigation of Gender Incongruence study (19). To achieve a kappa value of 0.8 with a standard error of 0.1 at a 50% prevalence of hirsutism, a study group of 36 individuals was required.

### Statistics

All data were exported to data editor SPSS Version 28.0 (IBM SPSS Statistics for Windows, Armonk, NU: IBM Corp). Continuous variables were expressed as mean and standard deviation, discrete variables were expressed as median and interquartile range, and categorical data were summarized in percentages. To evaluate longitudinal changes in terminal hair growth and patient satisfaction, linear mixed models (LMMs) were employed, with months of GAHT as a fixed effect and a random intercept for baseline scores. The Mann–Whitney *U* test was performed to examine differences in the reduction of terminal hair between lasered and non-lasered women. Spearman rank-order correlations were performed to examine whether changes in body appreciation were associated with changes in hair density at each anatomical site. Moreover, Spearman rank-order correlations were employed to investigate the association between the 12-month change in testosterone concentration and the 12-month change in hair density.

## Results

In total, 16 transgender women and 20 transgender men were included. Nine individuals who were otherwise eligible were not included due to personal or practical reasons. The characteristics of the transgender women and men are depicted in, respectively, Tables 1 and 2. Two individuals were lost to follow-up at *t* = 1, however they did visit at *t* = 2. Conversely, 6 transgender women and 4 transgender men were lost to follow-up at *t* = 2, yet they did visit at *t* = 1. GAHT recorded at baseline (*t* = 0) corresponds to the treatment initiated immediately after the baseline visit, whereas GAHT at *t* = 1 and *t* = 2 indicates the treatment participants were receiving at those time points. Any changes in either FHT (Table 1) or MHT (Table 2) occurred between study visits. No transgender women underwent electrolysis treatment during the first year of GAHT.

**Table 1:** Characteristics of the transgender women

	Baseline (*n* = 16)	3 months GAHT (*n* = 15)	12 months GAHT (*n* = 10)
Age (y), median (IQR)	25 (20-28)		
Ethnical background, *n* (%)			
White/Caucasian	15 (94%)		
Black or African American	0 (0%)		
Asian	1 (6%)		
MENA	0 (0%)		
Hispanic or Latino	0 (0%)		
Other	0 (0%)		
BMI (kg/m^2^), mean (SD)	23.43 (1.58)	23.62 (1.70)	24.05 (1.52)
Estradiol, *n* (%)			
Transdermal (gel or patch)	3 (19%)	12 (80%)	4 (40%)
Oral	13 (81%)	3 (20%)	6 (60%)
Antiandrogens, *n* (%)			
Triptorelin	3 (19%)	5 (33%)	8 (80%)
Decapeptyl	13 (81%)	10 (67%)	2 (20%)
Permanent hair removal methods, *n* (%)			
Laser, *n^a^* (%)	0 (0%)	5 (33%)	4 (40%)
0-5 treatments	0 (0%)	5 (100%)	0 (0%)
5-10 treatments	0 (0%)	0 (0%)	2 (50%)
10-15 treatments	0 (0%)	0 (0%)	1 (25%)
>15 treatments	0 (0%)	0 (0%)	1 (25%)
Estradiol concentration (pmol/L), median (IQR)	72 (51-100)	251 (192-505)	298 (161-396)
Testosterone concentration (nmol/L), median (IQR)	16 (12-22)	1 (1-1)	1 (1-1)

Abbreviations: MENA, Middle Eastern or North African

^*a*^Number (%) of total.

**Table 2:** Characteristics of the transgender men

	Baseline (*n* = 20)	3 months GAHT (*n* = 19)	12 months GAHT (*n* = 16)
Age (y), median (IQR)	21 (20-22)		
Ethnical background, *n* (%)			
White/Caucasian	15 (75%)		
Black or African American	0 (0%)		
Asian	3 15%)		
MENA	0 (0%)		
Hispanic or Latino	2 (10%)		
Other	0 (0%)		
BMI (kg/m^2^), mean (SD)	24.53 (1.24)	25.48 (1.34)	25.23 (1.34)
Testosterone, *n* (%)			
Transdermal (gel)	17 (85%)	13 (68%)	5 (31%)
Intramuscular	3 (15%)	6 (32%)	11 (69%)
Estradiol concentration (pmol/L), median (IQR)	197 (62-377)	86 (68-235)	128 (66-196)
Testosterone concentration (nmol/L), median (IQR)	1 (1-2)	15 (11-18)	13 (9-24)

Abbreviations: MENA, Middle Eastern or North African

After 3 months of GAHT, mean testosterone concentrations significantly increased with 15.26 nmol/L (95% CI 11.46-19.06, *P* < .001), while they decreased in transgender women with −13.40 nmol/L (95% CI −18.65 to −8.15, *P* = .002). Moreover, mean estradiol concentrations significantly decreased in transgender men after 3 months of MHT (−77.32 pmol/L, 95% CI −178.79-24.16, *P* = .040), while it significantly increased in transgender women with 259.60 (95% CI 154.18-365.02, *P*=<.001). Between 3 and 12 months of GAHT, no significant differences in hormone concentrations were observed for both transgender men and women.

### Longitudinal changes in terminal hair density by digital microscopy

**Figure 1:**
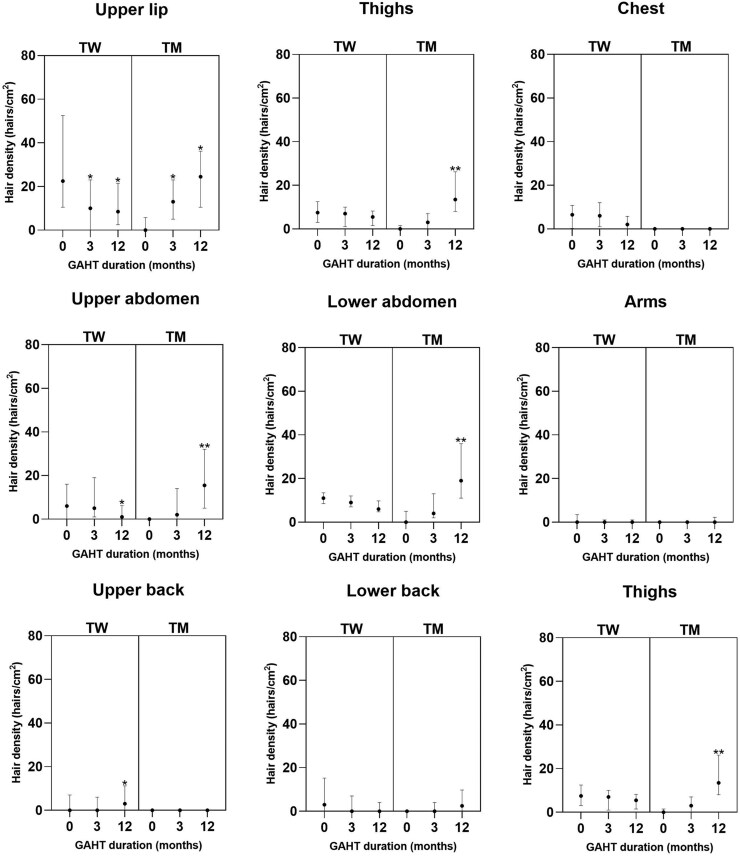
Median hair densities per location over time. TW = transgender women; TM = transgender men. Error bars represent the 90% confidence interval around the median.

Figure 1 shows the changes in hair density at the different locations, as determined using digital microscopy. In the total transgender women group, a significant decrease in hair density was already found after 3 months of FHT on the upper lip (−16 hairs/cm^2^ 95% CI −31 to −1, *P* = .033) and chin (−21 hairs/cm^2^ 95% CI −41 to −1, *P* = .039). After 12 months of FHT, hair density was significantly decreased on the upper lip (−17 hairs/cm^2^ 95% CI −32 to −2, *P* = .031), chin (−28 hairs/cm^2^ 95% CI −47 to −8, *P* = .008), and upper abdomen (−6 hairs/cm^2^ 95% CI −12 to 0, *P* = .034). The Spearman rank correlation revealed a poor association between the 12-month change in testosterone concentration and the 12-month change in chin hair density in transgender women (ρ=0.128, *P* = .724).

No statistically significant differences were observed in the decrease in hair density on the upper lip between transgender women who had undergone laser treatment and those who had not after 3 months of FHT (*U* = 19.5, *Z* = −0.675, *P* = .513) or after 12 months (*U* = 7.00, Z = −0.735, *P* = .556). The corresponding effect sizes suggest a small effect of laser treatment after 3 months(*r* = 0.174) and a small to moderate effect at 12 months (*r* = 0.232). Similarly, no significant differences were observed in the reduction of hair density on the chin between the groups after 3 months (*U* = 14.0, *Z* = −1.353, *P* = .206) or 12 months of FHT (*U* = 8.00, *Z* = −0.490, *P* = .730). However, the effect sizes indicate a moderate effect of laser treatment at 3 months (*r* = 0.349) and a small effect at 12 months (*r* = 0.155).

In transgender men, hair density on the upper lip showed a significant increase already after 3 months of MHT (12 hairs/cm^2^ 95% CI 4.7-19, *P* = .002). After 12 months of MHT, hair density was significantly increased on the upper lip (21 hairs/cm^2^ 95% CI 11-30, *P* = .002), chin (13 hairs/cm^2^ 95% CI 3-24, *P* = .013), upper abdomen (17 hairs/cm^2^ 95% CI 8-23, *P* < .001), lower abdomen (20 hairs/cm^2^ 95% C 11-28, *P* < .001), and thighs (14 hairs/cm^2^ 95% CI 7 to 21, *P* < .001). No differences in terminal hair density were found on the arm, neither for transgender women nor transgender men. The same accounts for the chest and upper back in transgender men. Two out of 16 individuals (12.5%) showed a response of <10 hairs/cm^2^ on the face (eg, upper lip and chin) after 12 months of MHT. Comparable to transgender women, in transgender men, the Spearman rank correlation showed a poor association between the 12-month change in testosterone concentration and the 12-month change in chin hair density (*ρ*=0.250, *P* = .350).

**Figure 2:**
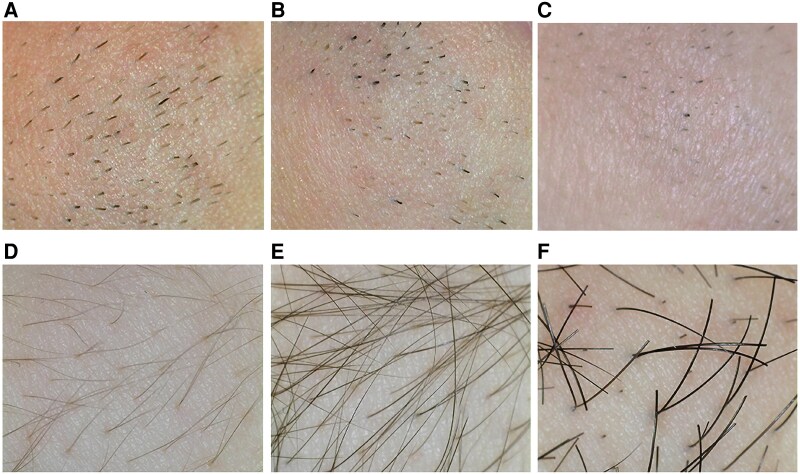
Morphologic changes in terminal hair growth by digital microscopy. A) upper lip of transgender woman at baseline (A), after 3 months of FHT (B), and after 12 months of FHT (C); upper abdomen of transgender man at baseline (D), after 3 months of MHT (E), and after 12 months of MHT (F).

Figure 2 visually shows gradual morphological changes in hair structures induced by GAHT. Figure 2A, 2B and 2c shows an example of terminal hair growth on the upper lip at, respectively, baseline, and after 3 months and 12 months FHT, without interference of laser hair removal treatments. After 3 months of FHT, a visible reduction in terminal hair density is already apparent, with a more pronounced decrease observed after 12 months. Figure 2D, 2E and 2f shows an example of the terminal hair growth at, respectively, baseline, and after 3 months and 12 months of MHT. After 3 months of MHT, hair pigmentation has notably increased, although the majority of hairs remained thin and fell within the vellus category. After 12 months (Fig. 2F), however, the hairs became more pigmented and visibly thicker, meeting the criteria for terminal hair.

### Patient satisfaction: general body image

Table 3 shows mean BAS-2 scores in transgender men and women. Among transgender women, body appreciation increased over time, with mean scores of 2.71 (SD 0.49) at baseline, 3.07 (SD 0.52) after 3 months, and 3.46 (SD 0.67) after 12 months of FHT, with a mean difference of 0.75 (95% CI 0.23-1.3, *P* = .008) between baseline and 12 months of FHT. A similar pattern was observed among transgender men. Their mean BAS-2 score increased from 3.41 (SD 0.74) at baseline to 3.51 (SD 0.62) after 3 months, and 3.96 (SD 0.60) after 12 months of MHT. The mean difference between baseline and 12 months of MHT was 0.56 (95% CI 0.11-1.0, *P* = .017).

**Table 3:** Body appreciation (BAS-2 scores) for transgender women and transgender men over time after start of GAHT treatment

BAS-2 item	Baseline		3 months GAHT		12 months GAHT	
	Transgender men	Transgender women	Transgender men	Transgender women	Transgender men	Transgender women
	Mean (SD)	Mean (SD)	Mean (SD)	Mean (SD)	Mean (SD)	Mean (SD)
I respect my body	3.75 (0.58)	3.85 (1.04)	3.80 (0.86)	4.00 (0.75)	3.90 (0.57)	4.38 (0.72)
I feel good about my body	2.31 (0.60)	2.80 (1.06)	2.60 (0.63)	3.21 (0.79)	3.50*^,#^ (0.71)	3.87*^,#^ (0.72)
I feel that my body has at least some good qualities	3.56 (0.73)	4.20 (0.89)	3.80 (0.68)	4.37 (0.83)	4.00* (0.67)	4.37 (0.62)
I take a positive attitude toward my body	2.50 (0.97)	3.20 (1.06)	2.80 (0.68)	3.37 (1.12)	3.30* (0.82)	4.00* (0.82)
I am attentive to my body's needs	3.56 (0.73)	4.15 (0.49)	3.80 (0.94)	3.84 (0.77)	3.90 (0.74)	4.06 (0.57)
I feel love for my body	2.06 (0.68)	2.95 (1.00)	2.53 (0.74)	3.11 (0.88)	3.00* (1.05)	3.69* (0.87)
I appreciate the different and unique characteristics of my body	2.37 (0.81)	3.45 (0.87)	2.80 (0.86)	3.63 (0.96)	3.10 (0.99)	3.81 (0.83)
My behavior reveals my positive attitude towards my body; for example: I hold my head high and smile	2.50 (0.89)	3.45 (1.00)	3.07 (1.10)	3.21 (0.79)	3.50*^#^* (1.08)	3.81* (0.66)
I am comfortable in my body	2.31 (0.79)	2.55 (1.15)	2.62 (0.51)	2.74 (0.93)	3.20*^,#^ (1.32)	3.69 (1.01)
I feel like I am beautiful even if I am different from media images of attractive people (eg, models, actresses/actors)	2.19 (0.66)	3.45 (1.10)	2.73 (0.8)	3.58 (0.84)	3.20 (0.80)	4.00* (0.97)
Total	2.71 (0.49)	3.41 (0.74)	3.07 (0.52)	3.51 (0.62)	3.46* (0.67)	3.96* (0.60)

Mean scores per item reflect responses to the Dutch validated version of the BAS-2 (18), administered at 3 time points (baseline, after 3 months, and 12 months of GAHT).

1: Never; 2: Seldom; 3: Sometimes; 4: Often; 5: Always.

*P < .05 across time vs. baseline. #*P* < .05 across time vs. 3 months of GAHT.

At baseline, 88% of transgender women stated that too much hair on the upper lip and chin was considered most burdensome, while 80% of transgender men stated that too little hair on the upper lip and chin was considered most burdensome. A non-parametric Spearman correlation was used to examine the association between changes in hair density and changes in body appreciation. However, no significant Spearman correlations were found in transgender women for the upper lip (*ρ* = −0.585, *P* = .075) or the chin (*ρ* = −0.256, *P* = .475), and none were found in transgender men either (upper lip: *ρ* = 0.012, *P* = .965; chin: *ρ* = 0.026, *P* = .923). The same accounts for the chest, upper and lower abdomen, arms, upper and lower back, and thighs.

### Patient satisfaction: hair growth pattern

Table 4 depicts patient satisfaction with hair growth per location over time. Among transgender women, overall mean satisfaction increased from 1.88 (SD 0.66) at baseline to 2.62 (SD 0.78) after 3 months, to 2.85 (SD 0.90) after 12 months of FHT, with a mean difference of 0.97 (95% CI 0.27-1.67, *P* = .010) between baseline and 12 months of FHT. Thus, after 12 months, mean total satisfaction increased from “Somewhat dissatisfied” to “Neither satisfied nor dissatisfied”. In transgender men, overall mean satisfaction increased from 3.21 (SD 0.72) at baseline to 3.47 (SD 0.58) after 3 months, and to 4.04 (SD 0.49) after 12 months of MHT, with a mean difference of 0.84 (95% CI 0.43-1.25, *P* < .001) between baseline and 12 months of MHT. This corresponds to a 12-month change from “Neither satisfied nor dissatisfied” to “Somewhat satisfied”. There were no significant differences in patient satisfaction between responders vs non-responders for the upper lip (1.21 95% CI −0.51-3.02, *P* = .086) nor the chin (0.5 95% CI −1.51-2.51).

**Table 4:** Satisfaction with hair growth per location over time

	Baseline		3 months GAHT		12 months GAHT	
	Transgender men	Transgender women	Transgender men	Transgender women	Transgender men	Transgender women
	Mean (SD)	Mean (SD)	Mean (SD)	Mean (SD)	Mean (SD)	Mean (SD)
Upper lip	1.25 (0.78)	2.35 (1.13)	2.00 (1.25)	3.00 (0.88)	2.90*^a^* (1.45)	3.56*^a^* (1.15)
Chin	1.25 (0.78)	2.15 (1.04)	1.93 (1.39)	2.68 (0.82)	2.80*^a,b^* (1.55)	3.44*^a^* (1.21)
Chest	1.69 (1.01)	3.00 (1.12)	2.53 (1.13)	3.53*^a^* (1.22)	2.56*^a^* (1.24)	4.06 (0.77)
Upper abdomen	1.81 (1.11)	3.70 (1.08)	2.87 (1.25)	3.84*^a^* (1.07)	3.00*^a^* (1.33)	4.37*^a^* (0.81)
Lower abdomen	1.56 (1.09)	3.60 (1.00)	2.13 (0.99)	3.63 (1.01)	2.40*^a,b^* (1.43)	4.37 (0.81)
Arms	2.31 (1.01)	3.25 (1.07)	3.00 (1.00)	3.37 (0.96)	3.20*^a^* (1.03)	4.00*^a^* (1.10)
Upper back	2.94 (1.48)	3.85 (0.93)	3.53 (1.36)	3.84 (0.90)	2.90 (1.37)	4.19 (0.91)
Lower back	2.56 (1.46)	3.80 (1.01)	3.27 (1.39)	3.74 (0.99)	3.11 (1.45)	4.19 (0.91)
Thighs	1.56 (0.81)	3.15 (1.23)	2.33 (1.11)	3.58*^a^* (0.77)	2.60*^a,b^* (1.27)	4.19*^a^* (0.84)

Satisfaction score 1: Very dissatisfied; 2: Somewhat dissatisfied; 3: Not satisfied nor dissatisfied; 4: Somewhat satisfied; 5: Very satisfied.

^*a*^ *P* < .05 across time vs baseline.

^*b*^ *P* < .05 across time vs 3 months of GAHT.

The results of an additional subset of items about cognitions and emotions related to hair growth over time are depicted in Table 5. There is a significant increase in perceived congruence between body hair extent and gender identity for both groups after 12 months of GAHT (transgender women: 1.2, 95% CI 0.28-2.42, *P* = .015; transgender men: 1.15, 95% CI 0.45-1.85, *P* = .002).

**Table 5:** Cognition, emotion, and behavior related to hair growth over time

	Baseline		3 months GAHT		12 months GAHT	
	Transgender men	Transgender women	Transgender men	Transgender women	Transgender men	Transgender women
	Mean (SD)	Mean (SD)	Mean (SD)	Mean (SD)	Mean (SD)	Mean (SD)
I feel insecure about the hair growth on my body.	4.44 (0.73)	2.85 (1.09)	4.07 (0.88)	2.89 (0.88)	3.50 (1.08)	2.44*^a^* (0.51)
I experience the extent of body hair as distressing.	4.81 (0.41)	2.40 (0.88)	4.27 (0.70)	2.63*^a^* (0.83)	3.70*^a,b^* (0.95)	1.87*^a^* (0.62)
I worry about the extent of hair on my body.	3.75 (0.86)	2.15 (0.81)	3.00 (0.85)	2.42*^a^* (0.84)	2.90*^a^* (0.99)	1.69*^a,b^* (0.61)
I perceive the extent of my body hair as congruent with my gender identity.	1.19 (0.54)	2.35 (1.18)	1.47 (0.64)	2.37 (0.95)	2.40*^a,b^* (1.27)	3.50*^a^* (0.89)

Score 1: Never; 2: Seldom; 3: Sometimes; 4: Often; 5: Always.

^*a*^ *P* < .05 across time vs baseline.

^*b*^ *P* < .05 across time vs 3 months of GAHT.

## Discussion

This longitudinal study investigated changes in body hair determined using digital microscopy, and its subsequent effect on patient satisfaction with body hair growth and overall body image at baseline and after 3 and 12 months of GAHT. In transgender women, there was a significant decrease in hair density on the upper lip, chin, and upper abdomen, whereas hair density significantly increased in transgender men after 12 months of MHT on the upper lip, chin, upper and lower abdomen, and thighs. The increase in hair density in transgender men in those specific locations is consistent with previous studies suggesting that the face (eg, upper lip and chin), lower abdomen, and thighs show high androgen sensitivity (12, 20). Moreover, the findings of the current study align with those of a previous multicenter study, which reported a marked decrease in terminal hair in transgender women and a significant increase in transgender men after one year of GAHT, as measured by the mFG score (19).

Despite the clear hormonal effects of GAHT, manifested as a significant increase in testosterone concentrations in transgender men and a significant decrease in testosterone concentrations in transgender women, the correlation coefficients demonstrated poor associations between changes in testosterone concentration and hair density over 12 months. However, pronounced within-group changes were observed in both terminal hair growth and testosterone concentrations. This apparent disconnect may be due to several factors, including interindividual variation in androgen sensitivity, interindividual differences in the conversion rate of testosterone to DHT, the timing of blood sampling, differences in testosterone administration route, treatment adherence, interindividual pharmacokinetics, and regional variation in hair growth responsiveness. Together, these factors likely obscure a direct linear relationship between biochemical changes and physical or morphological outcomes.

Within the transgender women group, some heterogeneity was introduced by the fact that some individuals had undergone laser therapy while others had not. As FHT gradually reduces terminal hair growth, many transgender women additionally pursue laser treatment to accelerate or enhance the reduction of, particularly, facial hair (9). Persistent facial hair in transgender women is particularly salient because it is highly visible and strongly associated with male secondary sex characteristics. In our study, we observed small to moderate influences of laser treatment on hair density reduction based on the effect sizes. However, no statistically significant differences in terminal hair reduction were observed between lasered and non-lasered participants, which might be caused by the limited sample size of the respective subgroups. Future studies with larger cohorts could further clarify the potential contribution of laser therapy.

While the findings of this study offer valuable insights into the early dermatological changes that occur during GAHT, it is important to recognize that they do not reflect the maximum effect of GAHT yet. The ultimate effect of GAHT on hair growth is influenced by the type of therapy, individual genetic factors, and the baseline biological hair growth, in addition to the GAHT dosage (9). For transgender women, the maximum effect of FHT on hair growth is typically achieved after 3 years, whereas transgender men generally reach the maximum effect of MHT after 5 years (9, 21, 22). In the present study, the follow-up period was limited to 12 months. While this duration is sufficient to observe initial changes in terminal hair growth, it likely does not capture the full effect of GAHT. Consequently, a longer follow-up would likely reveal more pronounced changes in hair growth patterns, as well as in the psychosocial impact.

A notable strength of this study lies in the use of digital microscopy, which offers objective quantification of terminal hair based on a diameter threshold, in contrast to the semi-quantitative and subjective nature of the mFG score. Digital microscopy reduces observer bias and allows for a more precise measure of subtle changes over time. Additionally, digital microscopy enables terminal hair quantification within 48 hours after dry shaving without compromising data integrity, while an assessment of the extent of terminal hair using the mFG score ideally requires discontinuation of hair removal methods for 3-4 weeks prior to an appointment. Digital microscopy also has constraints, particularly its limited surface area coverage. This was evident in the chest region, where the mFG score includes both areolar (score 1) and sternal (scores 2-4) hair growth. However, the current study only captured the sternal area, which could have resulted in an underestimation of the total hair density on the chest. Capturing multiple images per anatomical site could help to overcome this limitation in future research settings or clinical practice. These practical considerations do not detract from the fact that quantitative assessment using digital microscopy is a valuable method that facilitates reliable monitoring of therapeutic strategies, such as investigating the effects of different treatment regimens, including hormone therapy, as well as interventions such as laser hair removal and electrolysis. Moreover, it reduces reliance on subjective scoring systems and facilitates the identification of subtle but clinically meaningful effects over time. This technology may therefore facilitate more precise and individualized monitoring of treatment outcomes in both research and clinical practice.

Beyond physical changes, this study also examined psychosocial outcomes, revealing that general body image improved in both transgender women and transgender men after 12 months of GAHT. However, improvements in body appreciation were not associated with changes in hair density. This implies that factors other than hair growth may have contributed to the perceived improvements in body image. Improvements in body image after 12 months of GAHT are consistent with psychosocial findings from previous studies conducted among transgender individuals undergoing GAHT (23, 24). MHT primarily leads to amenorrhea, clitoral enlargement, and voice deepening, whereas FHT most markedly results in breast development, softer and less oily skin, and a reduction in semen production, spontaneous erections, and testicular volume (25). Particularly, amenorrhea and clitoral enlargement in transgender men, and a reduction in semen production and spontaneous erections in transgender women are outcomes that correlate with patient satisfaction (23). Although changes in terminal hair did not seem to be a contributing factor to the improvements in body image, this study did find improvements in overall patient satisfaction with individuals' hair growth pattern, and congruence between body hair and gender identity.

Limitations of this study include the relatively small sample size in both groups and the limited ethnic diversity among participants. Furthermore, there was some loss to follow-up at both *t* = 1 and *t* = 2, which may have introduced bias, as individuals who did not attend all study visits could differ systematically from those who completed the study. These factors could impact the generalizability of the outcomes of this study. Strengths of this study include its longitudinal study design and the use of digital microscopy, which enabled objective quantification of hair density.

## Conclusion

After 12 months of GAHT, transgender women showed a significant reduction in terminal hair growth, as indicated by reduced hair density determined by digital microscopy on the upper lip, chin, and upper abdomen. In contrast, transgender men exhibited a significant increase in hair density on the upper lip, chin, upper and lower abdomen, and thighs. Alongside these objective changes, both groups reported improvements in general body image. Yet, the correlation of these subjective improvements and alterations in hair density was weak. Importantly, satisfaction with body hair and perceived congruence between body hair and gender identity increased in both transgender women and men following 12 months of GAHT. These results highlight the measurable effects of GAHT on terminal hair growth and its positive association with psychosocial outcomes in transgender individuals undergoing gender transition. Quantitative assessment of hair density is a valuable method that can support future research comparing the efficacy of different GAHT regimens.

## Data Availability

Some or all datasets generated during and/or analyzed during the current study are not publicly available but are available from the corresponding author on reasonable request.
